# Prevalence and factors associated with treatment and control of hypertension among adults with hypertension in Myanmar

**DOI:** 10.1093/inthealth/ihac047

**Published:** 2022-07-18

**Authors:** Ze Haung, Seo Ah Hong

**Affiliations:** Township Department of Public Health, Myitkyina Township, Kachin State, Myanmar; ASEAN Institute for Health Development, Mahidol University, Salaya, Phuthamonthon, Nakhon Pathom 73170, Thailand

**Keywords:** adults, control, hypertension, Myanmar, treatment

## Abstract

**Background:**

Due to a dearth in the number of studies conducted in low- and middle-income countries, this study aimed to identify the prevalence and determinants of the treatment and control of hypertension among patients with hypertension in Myanmar.

**Methods:**

This community-based cross-sectional study was conducted among 410 adults who were registered for hypertensive treatment in health centers in Myitkyina Township, Kachin State, Myanmar. Multiple logistic regression was used to identify the associated factors.

**Results:**

The prevalence of treatment and control of hypertension was 48.1% and 20.5%, respectively. The factors associated with treatment were age (OR=2.60 for 46–60 y and OR=2.29 for 61–70 y compared with 30–45 y), ethnicity (OR=1.87), monthly family income (OR=1.90), comorbidity (OR=2.33), knowledge (OR=2.63) and adherence to physical activity (OR=1.86). Controlled hypertension was associated with age (OR=3.03 for 46–60 y and OR=2.27 for 61–70 y compared with 30–45 y), education (OR=1.81), comorbidity (OR=1.67) and adherence to medication (OR=3.45).

**Conclusions:**

The prevalence of treated and controlled hypertension was relatively low in this study. To improve the prevalence of hypertension treatment and control in this study population, effective and culturally sensitive intervention programs under universal health coverage should be established with an emphasis on individuals with lower educational attainment and younger ages.

## Introduction

Hypertension is a major topic of concern in public health globally, as it can lead to heart diseases, stroke and chronic kidney diseases.^1^ Hypertension is a major contributor to global mortality; it is estimated to be associated with 19.2% of deaths (10.7 million) worldwide.^2^ The financial burden for uncontrolled cases of hypertension is too expensive to neglect. Uncontrolled hypertension is defined as systolic blood pressure ≥140 mmHg and diastolic blood pressure ≥90 mmHg.^3^ The annual global direct healthcare cost due to uncontrolled hypertension is estimated at US$372 billion, representing about 10% of the world's overall healthcare expenditure.^4^ In fact, reducing blood pressure (BP) in patients with hypertension is highly beneficial to prevent the development of its complications and death. It is estimated that effective BP control could avert 0.77 million deaths among patients at medium to high cardiovascular disease (CVD) risk worldwide.^5^

Despite the importance of controlling hypertension, its treatment and control rates remain unacceptably low across the globe, particularly in low- and middle-income countries (LMICs).^6^ The most recent global estimates suggested that 36.9% of those with hypertension receive treatment globally, while only 20% achieve BP control in LMICs compared with 42% in high-income countries (HICs).^6^ Studies also estimated that the rates of hypertension treatment and control have significantly improved in HICs, but they are still at a modest stage in LMICs.^6^ As a result, the risk of dying from hypertension is more than double in LMICs than in HICs.^6^

Pharmacological treatment is critical to control BP and to prevent the development of its complications. Randomized clinical trials have demonstrated that commonly available antihypertensive medicines significantly reduce the risk of CVD and all-cause mortality.^7^ Along with medical treatment, lifestyle modification, including self-care behaviors such as maintaining body weight, adopting a healthy diet, engaging in physical activity, avoiding smoking and moderating alcohol consumption, also play an important role in the prevention and control of hypertension.^8^ Studies revealed that self-care behavioral factors were associated with being treated and controlled.^9,10^ However, there are limited studies on the association of self-care behaviors with hypertension treatment and control in LMICs.

In an attempt to improve the treatment and control rates of hypertension, understanding the factors associated with them might provide meaningful insights on how to better control hypertension. Sociodemographic factors such as age, gender, education, ethnicity, family income and body mass index (BMI) influence treatment and control.^10–14^ Likewise, knowledge on hypertension and self-efficacy in managing hypertension as psychological factors are also associated with being treated and controlled.^15,16^ Patients with hypertension necessitate regular follow-up and long-term treatment; therefore, accessibility to healthcare services is highly important for its effective management, especially in developing countries.^17^ Both qualitative and quantitative studies have underlined that the accessibility to healthcare services such as distance to health centers, perceived healthcare cost and the satisfaction derived from healthcare services influenced treated and controlled hypertension.^17,18^

Myanmar, an LMIC, is situated in Southeast Asia with diverse ethnicities, cultures, food habits and dietary patterns.^19^ There are seven states (Kachin, Kayah, Kayin, Mon, Rakhine, Shan and Chin) and seven regions (Ayeyarwady, Bago, Magway, Mandalay, Sagaing, Tanintharyi and Yangon) in Myanmar. Non-communicable diseases (NCDs) including hypertension are becoming prevalent and emerging as major public health concerns, and stroke and ischemic heart disease are leading causes of death in Myanmar.^20^ According to a nationwide study, the prevalence of hypertension in Myanmar was considerably high at 26.4% in 2014,^21^ while the WHO estimated the prevalence of hypertension to be 23% in 2018.^22^ Although the WHO's estimate was slightly lower than that of the nationwide survey, it was still the second highest prevalence among member countries of the Association of Southeast Asia Nations.^22^ With urbanization, adoption of a westernized lifestyle and economic development in Myanmar, some risk factors for NCDs such as smoking, alcohol consumption, unhealthy food habits, physical inactivity and obesity are on the increase.^23^

Despite the high prevalence of hypertension, limited information regarding the treatment and control of hypertension is available in Myanmar. Few studies have attempted to explore the treatment and control of hypertension in Myanmar.^8,21^ Additionally, since 2014, those kinds of studies have not been conducted in Myanmar or at least not in Kachin State. There is also little information on the associated factors for the treatment and control of hypertension. Therefore, this study aimed to describe the treatment and control rates of hypertension and their associated factors, such as sociodemographic, psychological, self-care behavioral and accessibility to healthcare services, among patients with hypertension in Myitkyina Township, Kachin State.

Hence, the findings of this study will serve as baseline information for policymakers and health professionals to formulate effective interventions in the prevention and control of hypertension in Myanmar. It will help to achieve Myanmar's national strategic plan of reducing, to a reasonable extent, the unconditional probability of dying between the ages 30 to 70 y from major NCDs by 20% from 2017 to 2025.^24^ The results here will also help provide a foundation to achieve the Sustainable Development Goals to reduce 25% of NCD mortality by 2025 in Myanmar.^22^

## Methods and Materials

### Study design and participants

This cross-sectional community-based study was carried out in Myitkyina Township, Kachin State, Myanmar during April and May 2019. Myitkyina is the capital city of Kachin State, the northernmost state of Myanmar. The ethnic make-up of Myitkyina includes Kachin, Shan and Burma-dominant ethnic groups. Adults aged 30–70 y who were registered for hypertension treatment in health centers in Myitkyina during the past year were included. The sample size was estimated using a CI of 95%, an acceptable error of 5% and the prevalence of medication adherence is 50%.^25^ Based on the total estimated target population in the Township (N=2567), this calculation determined a required sample size of 336. The sample size was increased to 402, which accounts for 10% probable non-response.

A multistage cluster random sampling method was used in this study. Stratified by rural and urban places of residence, one center among two urban health centers and four centers among six rural health centers were randomly selected. Each urban health center has their catchment areas (wards), and so do rural health centers (villages). Three wards of the selected urban health center and three villages of each of the four selected rural health centers were randomly selected. In total, three wards (urban areas) and 12 villages (rural areas) were selected. Lastly, adults aged 30 to 70 y and registered for hypertensive treatment in health centers were identified through outpatient medical records and were randomly selected. Those who were seriously ill, who had cognitive impairment and who were pregnant with gestational hypertension, were excluded. With the exclusion of 10 patients who did not meet the inclusion criteria or who were not available on the day of data collection, a total of 410 (97.6%) patients voluntarily agreed to participate in the study.

Ethical approval was obtained from the Committee for Research Ethics (Social Sciences), Mahidol University, Thailand (No. 2019/070.0204) and Institutional Review Board, University of Public Health, Yangon, Myanmar (UPH-IRB-2019/Research/20). Prior to the study, we also obtained permission from Township Public Health Department and identified the sample and made appointments with participants in collaboration with local health personnel (public health practitioners and certified midwives) and community authorized persons. Trained researchers collected data via face-to-face interviews at the participants’ residences. The objectives and procedure of data collection were explained to the participants prior to the survey. They were also assured of the protection of their rights and agreement and were told that they could terminate participation at any time without prejudice. Written informed consent was obtained from each participant. All data were treated anonymously using study identification numbers.

### Measurement

Treatment and control of hypertension

Treatment of hypertension was assessed by the question ‘How many of the past 7 days did you take your blood pressure pills?’ The participants who responded that they took ≥1 d were counted as receiving treatment. Control of hypertension was defined as systolic BP <140 mmHg and diastolic BP <90 mmHg using the Seventh Report of the Joint National Committee (JNC 7) classification.^8^ BP was measured twice at an interval of 2 min in a sitting position after taking a rest for a minimum of 5 min using digital sphygmomanometers (OMRON HEM-8712). BP was determined by the average of those two measurements.

Sociodemographic factors and accessibility to healthcare services

The sociodemographic factors included were age, gender, marital status, occupation, ethnicity, education and monthly family income in Myanmar Kyats (MMK), place of residence, duration of hypertension, family history of hypertension, comorbidity and weight status. Weight status was defined using BMI. Each participant’s weight was measured by digital scales to the nearest 0.1 kg and height by wooden height measuring boards to the nearest 0.5 cm according to the WHO guidelines.^26^ BMI was classified into four categories according to the guidelines for Asian adults: underweight (BMI<18.5 kg/m^2^), normal weight (18.5 kg/m^2^≤BMI<23.0 kg/m^2^), overweight or obese (BMI≥23.0 kg/m^2^).^27^ In addition, distance and satisfaction to the nearest health center and perceived healthcare costs were also included.

Psychological factors

Knowledge of hypertension was measured by the Hypertension Knowledge Level Scale questionnaire.^28^ It was composed of 22 items; questions elicited information regarding patient knowledge on the definitions of hypertension, lifestyle, treatment, adherence to medication and complications of hypertension. Answer options included ‘True’, ‘False’ or ‘Don't know’. A correct answer received a score of 1 whereas the score was 0 for an incorrect answer or ‘Don't know’. The total score possible ranged from 0 to 22. Those with a total score of ≥18.0 were considered as having an adequate level of knowledge, while those with a total score of <18 were classified as having an inadequate level of knowledge.^28^ Perceived self-efficacy to manage hypertension was measured using the Perceived Self-efficacy to Manage Hypertension Scale questionnaire.^29^ It was composed of five items concerning the level of confidence in managing hypertension and the response options ranged from 1 (‘not confident at all’) to 10 (‘totally confident’). Of the total score (5 to 50), those having a mean score of ≥9 were classified as having a good level, while the scores of <9 were classified as a poor level of confidence. Cronbach's alpha was 0.68 for knowledge and 0.79 for self-efficacy in this study.

Self-care behaviors

Self-care behaviors were measured by Hypertension Self-Care Activity Level Effect questionnaires (H-scale) developed by Warren and Seymour to assess the self-care behaviors of patients with hypertension in healthcare settings and epidemiological surveys.^30^ It is a 29-item scale that has six subdomains, such as adherence to antihypertensive medication (two questions), healthy diet (11 questions), engagement in adequate physical activity (two questions), practicing proper weight management (10 questions), avoidance of tobacco use (two questions) and abstinence from harmful alcohol drinking (two questions). The cut-off points on this scale were according to the original study's classification.^30^ Details of these self-care behaviors in this study have been described elsewhere.^31^

### Statistical analysis

Descriptive statistics (frequency and %) were used to describe sample distributions. χ^2^ tests were conducted to examine the association between hypertension treatment and control variables, and independent variables. Variables that showed associations with the outcomes at a significance level of 0.1 were entered into a logistic regression model using the backward method to predict controlled and treated hypertension. The data were analyzed in SPSS version 23 (IBM, Armonk, NY, USA).

## Results

The sociodemographic characteristics, accessibility to healthcare services, psychological factors and behavioral risk factors of patients with hypertension are presented in Table 1. The mean age of the population was 55.4 (SD=11.0) y, and more than two-thirds of the population were women (76.6%), of Kachin ethnicity (58.1%), living with a spouse/partner (67.8%) and living in rural areas (81.2%) (Table 1). The prevalence of hypertension treatment and control among all participants was 48.1% and 20.5%, respectively.

**Table 1.** Sociodemographic factors associated with hypertension treatment and control among adults with hypertension

		Treatment		Control	
	n (%)	n (%)	p value	n (%)	p value
Total sample	410 (100.0)	197 (48.1)		84 (20.5)	
**Sociodemographic factors**					
Age group, y					
30–45	85 (20.7)	29 (34.1)	0.0123	8 (9.4)	0.0130
46–60	176 (42.9)	94 (53.4)		44 (25.0)	
61–70	149 (36.3)	74 (49.7)		32 (21.5)	
Gender					
Male	96 (23.4)	56 (58.3)	0.0212	15 (15.6)	0.1774
Female	314 (76.6)	141 (44.9)		69 (22.0)	
Marital status					
Living without a partner	132 (32.2)	56 (42.4)	0.1162	30 (22.7)	0.4388
Married/living with a partner	278 (67.8)	141 (50.7)		54 (19.4)	
Occupation					
Employed/self-employed	84 (20.5)	51 (60.7)	0.0074	21 (25.0)	0.4971
Farmer	176 (42.9)	71 (40.3)		33 (18.8)	
Dependent/housewife/other	150 (36.6)	75 (50.0)		30 (20.0)	
Ethnicity					
Kachin	238 (58.1)	100 (42.0)	0.0040	43 (18.1)	0.1532
Other	172 (42.0)	97 (56.4)		41 (23.8)	
Education					
Primary school –	241 (58.8)	103 (42.7)	0.0102	39 (16.2)	0.0099
Secondary school +	169 (41.2)	94 (55.6)		45 (26.6)	
Monthly income (MMK)					
Low (<100 000)	180 (43.9)	65 (36.1)	<0.0001	28 (15.6)	0.0411
Middle (100 001–250 000)	102 (24.9)	54 (52.9)		21 (20.6)	
High (>250 000)	128 (31.2)	78 (60.9)		35 (27.3)	
Place of residence					
Urban	77 (18.8)	51 (66.2)	0.0004	17 (22.1)	0.7013
Rural	333 (81.2)	146 (43.8)		67 (20.1)	
Duration of hypertension, y					
≤3	167 (40.7)	68 (40.7)	0.0138	29 (17.4)	0.1941
>3	243 (59.3)	129 (53.1)		55 (22.6)	
Family history of hypertension					
Yes	151 (36.8)	77 (51.0)	0.3622	36 (23.8)	0.1990
No	259 (63.2)	120 (46.3)		48 (18.5)	
Comorbidity					
No	240 (58.5)	97 (40.4)	0.0002	38 (15.8)	0.0055
Yes	170 (41.5)	100 (58.8)		46 (27.1)	
Body mass index, kg/m^2^					
Overweight and obese (≥23.0)	266 (64.9)	137 (51.5)	0.0570	50 (18.8)	0.2490
Normal and underweight (<23.0)	144 (35.1)	60 (41.7)		34 (23.6)	

Abbreviation: MMK, Myanmar Kyat.

Tables 1 and 2 show the association between independent variables and treatment and control of hypertension using χ^2^ tests. In univariate associations, factors associated with treatment were age (p=0.0123), gender (p=0.0212), occupation (p=0.0074), ethnicity (0.0040), education (0.0102), monthly income (p<0.0001), place of residence (p=0.0004), duration of hypertension (p=0.0138), family history (p=0.3622), comorbidity (p=0.0002), BMI (p=0.0570) and knowledge (p<0.0001), as well as adherence to medication (p<0.0001), physical activity (p=0.0030) and weight management (p=0.0763). For controlled hypertension, they were age (p=0.0130), education (p=0099), monthly income (p=0.0411), comorbidity (p=0.0055), knowledge (p=0.0809) and self-efficacy (p=0.0153), as well as adherence to medication (p<0.0001) and physical activity (p=0.0218).

**Table 2.** Accessibility to healthcare services, as well as psychological and self-care behavioral factors associated with hypertension treatment and control among adults with hypertension

		Treatment		Control	
	n (%)	n (%)	p value	n (%)	p value
**Accessibility to healthcare services**					
Distance from nearest health center					
<1 mile (1.609344 km)	232 (56.6)	118 (50.9)	0.1455	53 (22.8)	0.1727
1–5 miles (1.609344–8.04672 km)	152 (37.1)	64 (42.1)		24 (15.8)	
>5 miles (8.04672 km)	26 (6.3)	15 (57.7)		7 (26.9)	
Satisfaction based on healthcare services					
Completely	324 (79.0)	150 (46.3)	0.1680	62 (19.1)	0.1880
To some extent/not at all	86 (21.0)	47 (54.7)		22 (25.6)	
Perceived healthcare costs					
Cheap	308 (75.3)	150 (48.7)	0.7053	64 (20.8)	0.8329
Expensive	101 (24.7)	47 (46.5)		20 (19.8)	
**Psychological factors**					
Knowledge on hypertension					
Inadequate	225 (54.9)	86 (38.2)	<0.0001	39 (17.3)	0.0809
Adequate	185 (45.1)	111 (60.0)		45 (24.3)	
Self-efficacy to manage hypertension					
Poor	331 (80.7)	156 (47.1)	0.4459	60 (18.1)	0.0153
Good	79 (19.3)	41 (51.9)		24 (30.4)	
**Self-care behaviors**					
Adherence to medication					
Non-adherence (<14 scores)	311 (75.9)	98 (31.5)	<0.0001	44 (14.2)	<0.0001
Adherence (14 scores)	99 (24.2)	99 (100.0)		40 (40.4)	
Adherence to healthy diet					
Low/middle diet quality (<52 scores)	402 (98.1)	192 (47.8)	0.4087	83 (20.7)	0.5719
High diet quality (≥52 scores)	8 (2.0)	5 (62.5)		1 (12.5)	
Adherence to physical activity					
Non-adherence (<8 scores)	308 (75.1)	135 (43.8)	0.0030	55 (17.9)	0.0218
Adherence (≥8 scores)	102 (24.9)	62 (60.8)		29 (28.4)	
Adherence to avoidance of tobacco use					
Non-adherence (≥1 scores)	204 (49.8)	101 (49.5)	0.5557	44 (21.6)	0.5895
Adherence (0 score)	206 (50.2)	96 (46.6)		40 (19.4)	
Adherence to weight management					
Non adherence (<40 scores)	371 (90.5)	173 (46.6)	0.0763	75 (20.2)	0.6737
Adherence (≥40 scores)	39 (9.5)	24 (61.5)		9 (23.1)	
Avoidance of harmful alcohol drinking					
No (>14 scores [M] and >7 [W])	9 (2.2)	2 (22.2)	0.1169	1 (11.1)	0.4810
Yes (≤14 scores [M] and ≤7 [W])	401 (97.8)	195 (48.6)		83 (20.7)	

Abbreviations: M, men; W, women.

Factors having p<0.1 in the bivariate analyses were employed in multiple logistic regression analyses (Table 3). The factors associated with treatment were age (OR=2.60, 95% CI 1.45 to 4.67 for 46–60 y and OR=2.29, 95% CI 1.24 to 4.21 for 61–70 y compared with 30–45 y), ethnicity (OR=1.87, 95% CI 1.20 to 2.91), monthly family income (OR=1.90, 95% CI 1.11 to 3.24 for middle and OR=2.56, 95% CI 1.54 to 4.25 for high compared with low income), comorbidity (OR=2.33, 95% CI 1.51 to 3.61), knowledge (OR=2.63, 95% CI 1.71 to 4.06) and adherence to physical activity (OR=1.86, 95% CI 1.12 to 3.08). Controlled hypertension was associated with age (OR=3.03, 95% CI 1.32 to 6.99 for 46–60 y and OR=2.27, 95% CI 0.96 to 5.39 for 61–70 compared with 30–45 y), education (OR=1.81, 95% CI 1.08 to 3.02 for secondary school or higher compared with primary or less), comorbidity (OR=1.67, 95% CI 1.00 to 2.79) and adherence to medication (OR=3.45, 95% CI 2.03 to 5.88).

**Table 3.** ORs and 95% CIs of hypertension treatment and control among adults with hypertension

	Treatment^a^		Control^b^	
	OR	(95% CI)	OR	(95% CI)
**Sociodemographic factors**				
Age group, y				
46–60 vs 30–45	2.60	(1.45 to 4.67)	3.03	(1.32 to 6.99)
61–70 vs 30–45	2.29	(1.24 to 4.21)	N.S	
Gender (female)	N.S.		-	
Occupation				
Farmer vs employed/self-employed	N.S.		-	
Dependent/housewife/other vs employed/self-employed	N.S.		-	
Ethnicity (other vs Kachin)	1.87	(1.20 to 2.91)	-	
Education (secondary school + vs primary-)	N.S.		1.81	(1.08 to 3.02)
Monthly income				
Middle vs low	1.90	(1.11 to 3.24)	N.S.	
High vs low	2.56	(1.54 to 4.25)	N.S.	
Place of residence (urban)	N.S.		-	
Duration of hypertension (>3 y)	N.S.		-	
Comorbidity (yes)	2.33	(1.51 to 3.61)	1.67	(1.00 to 2.79)
Weight status	N.s.		-	
**Psychological factors**				
Knowledge (adequate)	2.63	(1.71 to 4.06)	N.S.	
Self-efficacy (good)	-		N.S.	
**Self-care behavioral factors**				
Adherence to medication (yes)	N.S.		3.45	(2.03 to 5.88)
Adherence to physical activity (yes)	1.86	(1.12 to 3.08)	N.S.	
Adherence to weight management (yes)	N.S.		-	

Abbreviation: N.S., not significant.

^a^The variables included in the model are age group, gender, occupation, ethnicity, education, income, place of residence, duration of hypertension, comorbidity, obesity status, hypertension knowledge, adherence to medication, adherence to physical activity and adherence to weight management.

^b^The variables included in the model are age, education, income, comorbidity, hypertension knowledge, self-efficacy, adherence to medication and adherence to physical activity.

## Discussion

The current study aimed to describe the prevalence of treated and controlled hypertension and their associated factors among patients with hypertension. This study revealed that less than half of the respondents (48.1%) were treated. Hypertension treatment in this sample was higher than the national data in 2014, which estimated it at 34.9%, and a study in Yangon in the same year revealed 40.1%.^21,32^ In Myanmar, the WHO-recommended Prevention of Essential Non-communicable Diseases clinics project was only launched in 2017.^33^ The purpose of these clinics is to enhance the prevention, screening and treatment of NCDs including hypertension. The reason for the lower treatment rate in the Yangon study than in ours might be because the Yangon study was conducted before that project was launched. The hypertension control rate among all patients with hypertension in our study is also considerably low at 20.5%, while among treated patients it was 40.8%. However, the Yangon study stated that it was 45.3% among treated patients with hypertension.^32^ The disparity in the control rates in our study and the Yangon study might be attributable to the fact that the calculation was performed based only on treated patients with hypertension in the Yangon study, but all the patients with hypertension were included in our study.

Our study also found that several sociodemographic factors were associated with treated and controlled BP. Evidence supports that ethnic disparities may affect hypertension prevention, treatment and control.^34^ Our study also found that other ethnics (Burmese, Shan, Chinese and Nepalese) combined were more likely to be under treatment than in Kachin ethnics. Consistently, several studies have also documented that ethnicity is one of the associated factors for being treated.^35,36^ Therefore, a culturally sensitive hypertension control program should be developed to reduce ethnic disparities in the treatment of hypertension.

Studies showed that individuals of an older age were more likely to be under treatment.^12,13^ Our study revealed that participants aged 46–60 and 61–70 y were more likely to be treated than those aged 30–45 y. This might be explained by participants being more sensitive to their health condition as they grew older and trying medicines to treat their diseases. This implies that younger individuals were less likely to be under treatment and they should be educated to seek treatment early to prevent hypertensive complications. Moreover, previous studies stated that older-aged individuals were more likely to have controlled BP.^13,14^ On the contrary, our study revealed that the respondents in middle age (aged 46–60 y) were more likely to have their BP controlled than younger individuals (aged 30–45 y), while no association was observed among older individuals (aged 61–70 y). One explanation may be that younger individuals had a lower frequency of control because they also had a low frequency of treatment. By contrast, while treatment was higher among older individuals, comorbid conditions or hypertensive complications could be influencing control. Moreover, our study also revealed that respondents with equal or more than secondary education level predicted controlled BP than those with equal or less than primary education level, which is supported by other studies.^11,14^ One potential explanation is that poor education attainment has been found to be associated with reduced levels of hypertension knowledge and poor compliance to hypertension management.^37,38^ These findings highlight the importance of educational attainment as a marker of better control of hypertension and point out that improving educational status may be a pathway for better hypertension management.^39^

Our study also showed that the likelihood of being treated was associated with higher family income, which is also consistent with other studies.^40,41^ Poor affordability of medicines for the prevention of cardiovascular complications was highlighted earlier in the Prospective Urban Rural Epidemiology study.^42^ In Myanmar, the most recent government health expenditure was only 4.79% of the gross domestic product in 2018.^43^ This is still a very low percentage and a majority of health spending (75%) is out-of-pocket spending by households.^24^ Hence, to minimize this affordability problem, Myanmar should establish a financial risk management program like universal healthcare coverage to reduce out-of-pocket expenditure on health.^44^ Moreover, comorbid participants had higher odds of being treated than those without comorbidity in our study, which is in line with other findings.^10^ It is possible that the respondents may have been more concerned about their diseases if they became comorbid. Thus, patients without comorbidity should be reminded not to take for granted the need to get treatment on time in order to prevent hypertensive complications. Our study stated that having comorbidity was one of the predictors of having controlled BP, which is concordant with a study in China.^14^ This might be explained by respondents with comorbidity being more aware of their health condition because they are comorbid and thus had better compliance to hypertension management and therefore their high BP was more likely to be controlled.

As a psychological factor, there is another meaningful association between having knowledge about hypertension and being under treatment in our study. In accordance with previous studies,^15,45^ it was stated that those respondents with an adequate level of knowledge about hypertension were more likely to be under treatment than those with inadequate levels of knowledge. Several studies have revealed that knowledge gaps are important barriers to the effective prevention and treatment of hypertension.^15,46^ Knowledge has also been acknowledged as an important factor for adoption and sustained health behaviors.^47^ However, fewer than half of the participants reported having adequate knowledge about hypertension in our study. Hence, to improve patients’ behaviors concerning getting treatment, they should be educated through an effective education program.

Several studies have identified the benefits of adopting self-care behaviors as aspects of lifestyle modifications in reducing high BP.^8^ As a self-care behavioral factor, our study also stated that adherence to engage adequate physical activity had higher odds of being treated. It seems that respondents who were more likely to engage in lifestyle modification also followed other positive lifestyle habits, including getting treatment.^48^ Furthermore, similar to other studies,^9,49^ this study also found that adherers to medication were more likely to have BP controlled than non-adherers. As hypertension is a chronic disease and needs long-term treatment, medication adherence is key to reducing high BP and preventing its complications.^50^ In addition, studies have stated that medication adherence was associated with lower rates of hospitalization and healthcare costs.^51^ Thus, patients with hypertension should be encouraged to constantly adhere to medication.

This study had some limitations. As a cross-sectional study, the findings cannot be used to establish a conclusive cause-and-effect relationship between independent and dependent variables. In addition, the findings of this study cannot be generalized to the entire country. Another limitation is that we did not study the list of medicines that the respondents were taking. If we had studied it, we would have estimated the prevalence of resistant hypertension in this population. However, this study had several strengths. Firstly, participants were selected based on their residences by multistage random sampling techniques, although they were limited to those registered in health centers. Secondly, we measured the respondents’ BP according to a standard guideline in their residences, which may prevent the bias of white coat hypertension that may interfere with the interpretation of the condition of controlled hypertension.^52,53^ Thirdly, because most of the previous studies were conducted in big cities with the dominant ethnic group in Burma, this study was conducted among minority ethnic groups in the northern part of Myanmar. Thus, it provides some insights into the treatment and control of hypertension among ethnic minorities.

## Conclusions

This study revealed that hypertension treatment and control among patients with hypertension were low in this multiethnic sample of adults from Myanmar. Moreover, there were associated factors with treatment and control that could be considered in the prevention and control of hypertension in this study population. Therefore, to improve treatment and control rates among patients with hypertension in this study area, effective and culturally sensitive intervention programs under a financial risk management program like universal health coverage should be established. That program should place an emphasis on individuals with lower educational attainment and younger age. For future studies, we recommend the study of other socioeconomic factors associated with low hypertension treatment and control, such as availability or accessibility to treatment, provision of free medicines, out-of-pocket expenses and costs of drugs, through qualitative and quantitative methods.

## Data Availability

The data underlying this article will be shared on reasonable request to the corresponding author.
